# An integrated modeling approach to estimating Gunnison sage-grouse population dynamics: combining index and demographic data

**DOI:** 10.1002/ece3.1290

**Published:** 2014-10-22

**Authors:** Amy J Davis, Mevin B Hooten, Michael L Phillips, Paul F Doherty

**Affiliations:** 1 Department of Fish, Wildlife, and Conservation Biology, Colorado State UniversityFort Collins, Colorado, 80523; 2 U.S. Geological Survey, Colorado Cooperative Fish and Wildlife Research Unit, Colorado State UniversityFort Collins, Colorado, 80523; 3 Colorado Parks and Wildlife317 W. Prospect Rd, Fort Collins, Colorado, 80526

**Keywords:** Bayesian, *Centrocercus minimus*, growth rate, integrated population model, lek counts, Leslie transition matrix, population projection

## Abstract

Evaluation of population dynamics for rare and declining species is often limited to data that are sparse and/or of poor quality. Frequently, the best data available for rare bird species are based on large-scale, population count data. These data are commonly based on sampling methods that lack consistent sampling effort, do not account for detectability, and are complicated by observer bias. For some species, short-term studies of demographic rates have been conducted as well, but the data from such studies are typically analyzed separately. To utilize the strengths and minimize the weaknesses of these two data types, we developed a novel Bayesian integrated model that links population count data and population demographic data through population growth rate (*λ*) for Gunnison sage-grouse (*Centrocercus minimus*). The long-term population index data available for Gunnison sage-grouse are annual (years 1953–2012) male lek counts. An intensive demographic study was also conducted from years 2005 to 2010. We were able to reduce the variability in expected population growth rates across time, while correcting for potential small sample size bias in the demographic data. We found the population of Gunnison sage-grouse to be variable and slightly declining over the past 16 years.

## Introduction

Information is frequently sparse for rare and declining species (Beissinger and McCullough 2002) and is often poor quality or has little inferential value (Engler et al. 2004; McKelvey et al. 2008). For bird species, large-scale count surveys, such as lek counts, often generate large amounts of data, but those data may be of questionable value (Walsh et al. 2004). However, for many species, the most extensive information available is from these types of surveys (Sauer et al. 1994). Therefore, a strong motivation to make the most of this type of data exists. Short-term demographic studies may also be conducted. These data are typically analyzed separately from long-term monitoring data, but uncertainty and possible bias can exist in these analyses especially if the study is not long enough to capture the range of annual variability present in the system (Bierzychudek 1999). Recent work has focused on using intensive, short-term demographic data to bolster information inherent in long-running, index data with integrated modeling approaches (e.g., Catchpole et al. 1998; Abadi et al. 2010b).

Integrated modeling approaches have been used on many bird species (Catchpole et al. 1998; Besbeas et al. 2002; Brooks et al. 2004; Gauthier et al. 2007; Abadi et al. 2010b) and several mammal species including bats (Schaub et al. 2007), seals (Besbeas et al. 2005; Thomas et al. 2005), and kangaroos (Chee and Wintle 2010). Previous research focused on using different types of survey data in integrated models including breeding bird surveys (Besbeas et al. 2002, 2003; Brooks et al. 2004), line transects (Chee and Wintle 2010), bat roost surveys (Schaub et al. 2007), and aerial surveys (Gauthier et al. 2007). Demographic data that have been combined with such survey data in integrated models include capture–recapture data (Gauthier et al. 2007; Schaub et al. 2007; Abadi et al. 2010a,b), ring-recovery data (Besbeas et al. 2002, 2003; Brooks et al. 2004), and reproductive success data (Schaub et al. 2007; Abadi et al. 2010b). Integrated modeling has been used to improve demographic and population parameter estimates (Brooks et al. 2004; Schaub et al. 2007; Abadi et al. 2010a), to evaluate population projections (Besbeas et al. 2002, 2005), to estimate immigration rates (Abadi et al. 2010b; Schaub et al. 2012), and to evaluate the effects of culling on population size (Chee and Wintle 2010).

Improving indices of temporal variation in relative abundance and evaluating population projections are two primary motivations for applying integrated population modeling to Gunnison sage-grouse (*Centrocercus minimus,* GUSG) data. GUSG have declined substantially from their historic numbers and range (Schroeder et al. 2004; Gunnison Sage-Grouse Rangewide Steering Committee 2005) and are proposed endangered under the U.S. Endangered Species Act (U.S. Fish and Wildlife Service, U. 2013). GUSG demographic rates have been evaluated using capture–recapture methods from 2005 to 2010 (Davis 2012). Population projections for this species suggest the population is currently declining (Davis 2012); however, the demographic data that produced this projection are based on a relatively small time frame of only 6 years.

Long-running population index data (i.e., lek counts) have been collected on GUSG since 1953 (Colorado Parks and Wildlife, CPW, unpublished data). Like many grouse species, the GUSG are a lekking species; males congregate on open tracts of land to strut and display for breeding opportunities with females. Counting males on leks provides a reliable opportunity to survey this typically elusive species (Patterson 1952; Rogers 1964). Although over 60 years of GUSG lek count data are available, the utility of these data as an abundance index is uncertain (Emmons and Braun 1984; Connelly et al. 2003; Walsh et al. 2004). Some of the main concerns with lek count data come from the lack of protocol standardization for many studies, lack of consistency between number of leks counted per year, the high level of within-year variation in lek count data (which may lead to large variance and potential bias), and the lack of accounting for detectability (Walsh et al. 2004).

Previous work on integrated modeling has combined data types similar to the data available for the GUSG (capture–recapture data and count data). However, the dominant method that has been used for these analyses is to combine a transition matrix of demographic data directly into a state-space model that computes the population size at each time step. This method assumes a relationship between the data sources, but allows for estimation or sampling error between the projections. GUSG count data are not applicable to this method because the count data available are an index to only the number of males in the population and a large number of strong assumptions need to be made to use it as an index to population size (e.g., a same proportion of the males are always present on the lek at the time of the high count; the sex ratio is constant and known).

We developed a novel methodology that links two data sources, with no direct parameters in common, through a derived parameter (*λ*, population growth). We acknowledge these parameters are not necessarily identical, but they are likely related as they are measures of the same population metric. We were able to model this relationship between growth rate estimates, which adds flexibility to the technique. We demonstrate the utility of this approach by modeling GUSG population growth rate. Using a Bayesian framework, we combine the more rigorous demographic data with the long-term population index data to evaluate population growth rate and compare hypotheses about trends in growth rate over time.

## Methods

### Study area

GUSG are distributed into seven isolated populations in southwest Colorado and the eastern part of Utah. The data included in this analysis were collected in the largest of these populations, Gunnison Basin. Over 85% of the existing individuals are thought to be in the Gunnison Basin population in Gunnison and Sagauche Counties, Colorado, USA (Gunnison Sage-Grouse Rangewide Steering Committee 2005).

### Data

**Figure 1:**
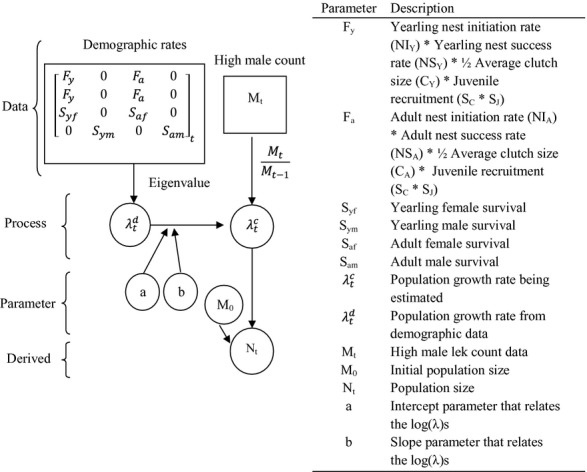
Directed acyclic graph of the structure of the integrated population model for Gunnison sage-grouse. Estimated parameters are represented by circles, and data are represented by squares. Demographic rates are in a prebreeder population matrix model.

The two data types that have been collected on GUSG are population demographic data (e.g., survival and reproduction) and population survey data consisting of high male counts on leks. The demographic data were collected using mark–recapture and radio telemetry methods (full details in Davis 2012). Adult and yearling birds were trapped in the spring from 2005 to 2010 and fitted with necklace-styled radio transmitters (Advanced Telemetry Systems, Inc., Isanti, MN). Females were tracked daily through the breeding season to determine nesting status and nest fates. Within 24–48 h of a nest hatching, researchers caught the resulting chicks and tagged them with radio transmitters (Advanced Telemetry Systems, Inc., Isanti, MN). Chicks were tracked daily until the fall (>60 days of age). During the fall and winter, all birds were tracked monthly using aerial telemetry. Nest success, chick, and adult survival rates were analyzed using known-fate models in Program MARK (White and Burhnam 1999). Accordingly, data were included for individuals only during the time frame in which their fates were known. Known-fate models allow for staggered entry (Pollock et al. 1989), as well as unequal sampling intervals. Individuals that go missing or those that slipped their collars were included as alive in the study area until their last encounter and then were right censored from the study. Demographic rates of fecundity and survival were estimated from this data. Fecundity rates were known only for females, and thus, we used a female-based population model. The female reproductive parameter F includes as follows: the nest initiation rate, the probability the female will have a successful nest (including renests), average clutch size, and juvenile recruitment (including daily chick survival to 30 days of age and juvenile monthly survival; equations given in Fig. 1). Female reproductive rates for both yearlings (*F*_*y*_) and adults (*F*_*a*_) and survival for yearling males (*S*_ym_), yearling females (*S*_yf_), adult males (*S*_am_), and adult females (*S*_af_) were estimated from 2005 to 2010 in Davis (2012, Appendix S1). The estimated means, variances, and covariances between the vital rates were calculated in Davis (2012, Appendix S2).

**Figure 2:**
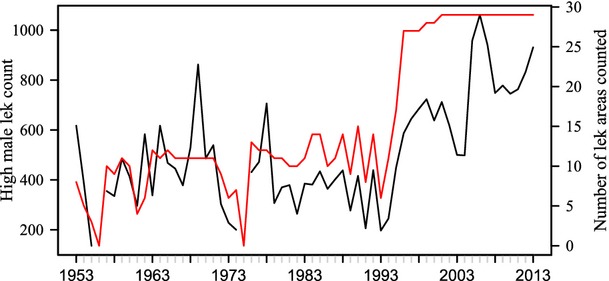
Plot of high male lek counts by year (black line with corresponding axis on the left) and number of lek areas counted over time (red line corresponding axis on the right) for Gunnison sage-grouse in Gunnison Basin, Colorado, USA (data provided in Appendix S4).

Population survey data used in this analysis come from lek counts in Gunnison Basin, Gunnison County, Colorado from 1953 to 2012 (Fig. 2). General protocols for lek counts are that all known leks are counted around 3 times during the spring lekking season, observers count the number of males on the lek from an hour before sunrise until the birds disperse, and the maximum number of males counted at any time on the lek is the data used in lek counts. Lek count data (*M*_*t*_) were not collected for 2 years (1956 and 1975). For these 2 years where no data exist, we used the average of the two adjacent years as imputations. Most of the lek count data available are not by individual leks but by lek areas (a collection of leks in relative proximity to each other). The number of lek areas surveyed (*l*_*t*_) annually was not consistent for the first ∼40 years that data were collected and averaged around 10 lek areas counted per year. In the last 17 years, the number of lek areas counted has been more consistent and averaged around 30 (Fig. 2). This greater consistency in survey effort was the result of the protocol being standardized in the mid-1990's, which regulated the number of individual leks surveyed, how often leks were surveyed, and when and how counts were conducted (Gunnison Sage-Grouse Rangewide Steering Committee 2005). We first fit the integrated model over the time period 1996–2012 (when the protocol was standardized). We then fit the model to the entire data set (1953–2012) to evaluate the methodology under more variable data.

### Integrated population model

When the demographic data (e.g., survival and reproduction rates) are arranged in a Leslie-type population matrix (equation 1), population projections can be made and vital rate sensitivities can be calculated (Caswell 2000, 2001). The population matrix model used here is a prebreeding matrix, and therefore, there are two age classes (yearling and adult). The population growth rate is calculated as the dominant eigenvalue from Leslie matrices (Caswell 2001). This is the growth rate when the population is at the stable age distribution. The stable age distribution is the dominant eigenvector (Caswell 2001).


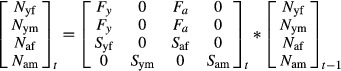
(1)

The lek count data are an index of population size, and population growth rate can be estimated as *λ*_t_ = *M*_*t*_/*M*_*t*-1_, (where *M*_*t*_ is the high male count at time *t*). Thus, the population metric that both data types can estimate is the population growth rate (*λ*). Although these growth rates may not be identical, they are certainly related as they are both measures of population change.

The core of our integrated specification model is a Malthusian growth model on the lek count data (Malthus 1798; Savage et al. 2004). The Malthusian growth model involves the rate of population change based on the lek count data (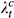, equations 2 and 4). As the lek counts do not have equal sampling effort, we included a weighting variable (*ω*_*t*_) in the Malthusian growth model; this variable adjusts the count based on the proportion of the total number of lek areas that were counted in each year (equation 3). The population growth rate (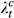) is assumed to be log-normally distributed (thus, 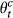 is normally distributed) with mean (*μ*_*c*_) and variance (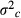; equation 6). We used conjugate priors for the mean (*μ*_*c*_) and variance (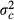; equations 7 and 8).


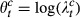
(2)


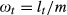
(3)


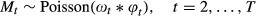
(4)


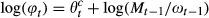
(5)


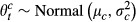
(6)


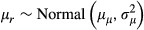
(7)


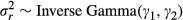
(8)

We modeled the growth rate from the demographic data (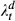) as log-normally distributed (thus, 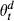 is normally distributed, based on equation 9) with a mean representing the relationship with the log of the lek count growth rate (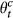, equation 10). The variance parameter for this relationship was modeled with an inverse gamma prior (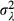, equation 11). To account for a potential bias between the two growth rates, we modeled the intercept parameters in the linear equation (*a*) as normally distributed centered at zero (equation 12). The lek data are male based; the population matrix model is female focused (as reproduction is based solely on female contribution). Therefore, the growth rates may not be identical. To estimate this possible difference, we modeled the slope parameter in the linear equation with a normal distribution centered at 1 (*b*, equation 13). The benefit of this model formulation is that it directly relates the two data types in a single common parameter, growth rate (Fig. 1).


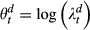
(9)


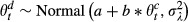
(10)


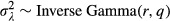
(11)


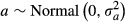
(12)


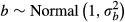
(13)

The prior values for *μ*_*c*_ were selected to be relatively flat and centered at zero, representing a stable population (Table 1). The priors for 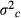 were chosen to improve the predictability of the model. This was accomplished by assessing the model fit for varying values of 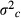 using a Bayesian *P*-value of MSE to ensure the estimated 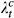 values had a similar variability to the observed data. The demographic data are statistically stronger and more reliable data; therefore, we selected strong priors for 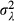 to reflect greater confidence in the demographic data (Table 1). The priors for the variance parameters around *a* and *b* were kept tight as there were little data to inform this relationship (Table 1).

**Table 1:** Prior values used in the integrated model on Gunnison sage-grouse

Prior is for the distribution of	Prior parameter	Value used for 1996–2012 data set	Description
*μ*_c_	*μ*_*μ*_	0.02	Mean for the mean distribution of log (*λ*)
	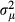	0.50	Variance for the mean distribution of log (*λ*)
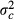	*γ*_1_	9.9	Shape parameter for the distribution of variance of log (*λ*)
	*γ*_2_	102	Scale parameter for the distribution of the variance of log (*λ*)
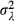	*r*	999	Shape parameter for the variance between the two *λ*s
	*q*	2.001	Scale parameter for the variance between the two *λ*s
*a*	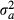	0.001	Variance for the intercept parameter relating the two *λ*s
*b*	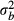	0.001	Variance for the slope parameter relating the two *λ*s

Of particular interest for a species of concern is how the population dynamics are changing over time. We compared three models in terms of the average growth rate and how it might be changing with time as follows: (1) Growth rate is constant across time, (2) Growth rate changes linearly with time, and (3) Growth rate has a quadratic relationship with time. We calculated the Watanabe–Akaike information criterion (WAIC) for each model to compare their relative support (Watanabe 2010; Hooten and Hobbs 2014). WAIC is a purely Bayesian method for comparing models and is similar to Akaike's information criterion (AIC) in that smaller values suggest more parsimonious models.

To calculate the posterior distribution for the parameters of interest, we fit the integrated model using a MCMC algorithm written in R (version 2.15, R Development Core Team 2012, code in Appendix S3). Diagnostic plots suggest that convergence occurred within 500 iterations for most parameters. We ran 20,000 iterations of the MCMC algorithm and discarded the first 2000 iterations as burn-in.

### Population projections

In order to obtain population estimates, we used a state-space model to calculate the population size at each time step based on a Leslie transition matrix (equation 14).


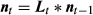
(14)

However, we only had demographic data available from 2005 to 2010. Therefore, in order to populate the state-space model for the rest of the timeline, we generated 10,000 sets of vital rates from a logit transformation of a multivariate normal distribution to allow for covariance between the vital rates based on the means and variances observed in the 6-year data set (methods described in Davis 2012). The corresponding growth rate (*λ*) values were calculated for each set of simulated vital rates. We matched estimated posterior predicted growth rate values at each time step from the integrated model to growth rate values from simulated vital rate values. Thus, when demographic data were not available, we selected the set of vital rates that corresponded to the growth rate that minimized the difference to the growth rates from the posterior predictive distribution of the integrated model.

The 6 years of demographic data indicated a period of decline (based on both the demographic estimates themselves and the lek count data, Fig. 2). Based on the lek count information, the range of growth rates is likely greater than that created from simulated data based on these 6 years of demographic information. Therefore, the *λ* matching strategy we used to calculate population sizes is likely to be biased low because the simulated vital rates do not experience growth rates as high as the lek count data suggest. In order to adjust for this, yet still maintain the correlation structure for the simulated vital rates, we multiplied the covariance structure from the 6 years of demographic data by a constant to increase the range of growth rates that can be achieved by this simulation method. We used graphical checks on the posterior predictive estimates compared to the growth rate used from the simulated set to ensure the simulated data were representing the range of values expected.

The population projections rely on an initial population size (*M*_o_). We used a Poisson distribution with mean *β*_o_ to generate the initial population size (equation 15).


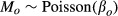
(15)

Previous research on sage-grouse species has found that typically between 42% and 67% of males are on a lek during a lek count (Walsh et al. 2004; Gunnison Sage-Grouse Rangewide Steering Committee 2005; Stiver 2007). Additionally, studies suggest that there is a 1.6:1 female to male ratio for sage-grouse (CPW unpublished report, Gunnison Sage-Grouse Rangewide Steering Committee 2005). For the model fit to the 1996–2012 data, we used the average lek count from 1996 to 2012 (i.e., 725) multiplied by the population correction factor (i.e., 4.73; based on the expected ratio of male lek counts to the expected population size) to obtain an estimate of the mean of the Poisson distribution, *β*_o_, for the initial population size (i.e., 3429).

## Results

**Figure 3:**
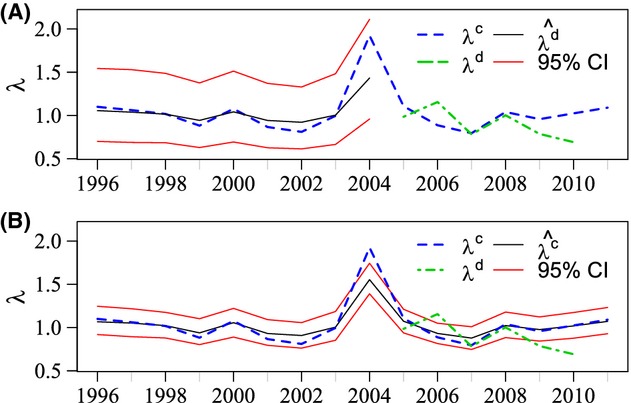
(A) Posterior predicted values for the population growth rate from the demographic data (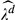) when demographic data were not available (1996–2004). (B) Posterior means for the population growth rate from the lek count data (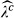). Both for data set from 1996 to 2012 with 95% credible intervals. The growth rates calculated from the lek count data (*λ*^c^, from 1996–2012) and demographic data (*λ*^d^, from 2005 to 2010) are shown for comparison.

We compared three models that examine how growth rates for GUSG might be changing over time: a constant model, a linear trend model, and a quadratic trend model. Based on the Watanabe–Akaike information criterion (WAIC), the most parsimonious model is the constant model (Table 2), indicating the distribution from which the lek count growth rates arise is relatively stable during the time frame examined (Fig. 3, Table 3). The posterior estimate of growth rate from the top model suggests the population that is declining slightly but the credible intervals include a stable population (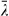 = 0.984, 95% CI: 0.879, 1.179).

**Table 2:** Watanabe–Akaike information criterion (WAIC) values comparing the three models on Gunnison sage-grouse population growth rate for the time frame from 1996 to 2012

Model	WAIC
Constant population growth rate	1743.9
Linear trend on population growth rate	1776.3
Quadratic trend on population growth rate	2815.7

Lower WAIC values denote a more parsimonious model. However, rules of thumb denoting model support in Akaike's information criterion (e.g., ΔAIC > 4 suggests little support) are not applicable as WAIC is a fundamentally different criterion. WAIC has a penalization element for the effective number of parameters as the actual number of parameters can be impossible to enumerate in hierarchical Bayesian models.

**Table 3:** Posterior means and 95% credible intervals for parameter values from the integrated model for Gunnison sage-grouse in Gunnison Basin, Colorado

			Posterior 95% Credible Interval	
Parameter	Description	Posterior Mean	Lower	Upper
*b*_0_	Mean parameter for the log *λ* from the lek count data	0.019	−0.002	0.042
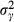	Variance parameter of the log *λ* values from the lek count data	0.003	0.002	0.004
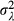	Variance parameter of the log *λ* values from the demographic data	0.033	0.010	0.087
*a*	Difference between log *λ* from lek count data and log *λ* from demographic data	−0.019	−0.077	0.039
*b*	Slope of the difference between log *λ* from lek count data and log *λ* from demographic data	0.811	0.631	0.951

**Figure 4:**
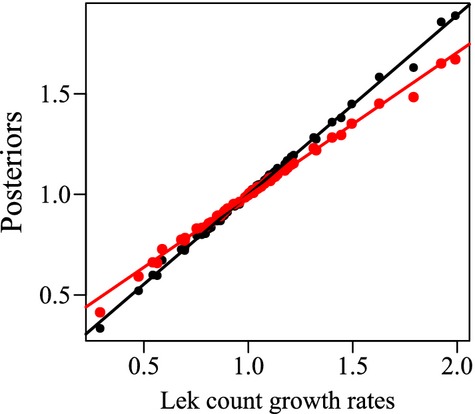
Raw lek count growth rates for Gunnison sage-grouse plotted against the posterior estimates for the lek count growth rates (black) and against the posterior predicted demographic growth rates (red). The lines show the linear relationship between the posterior estimates and the data. The tight fit of the black points shows how well the posteriors estimates fit the data.

The growth rates from the demographic data are generally lower than those from the lek count data (*a* = −0.019, 95% CI: −0.077, 0.039, Table 3). The slope between the growth rates (b) was estimated to be 0.811 (95% CI: 0.631, 0.951). The two data sources are positively correlated, but this value shows the index data fluctuate slightly more than the demographic data. By plotting the raw lek count growth rates against the posterior estimates for the lek count growth rates and against the posterior predicted growth rates for the demographic data, we can see how this relationship shows the stabilizing effect of the demographic data (Fig. 4).

The posterior estimates of the variance for the distribution that relates the lek count and demographic growth rates (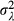) were 0.033 (95% CI 0.010, 0.087, Table 3). The variance estimate relating the lek count growth rates to the mean (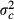) was 0.003 (95% CI: 0.002, 0.004). We conducted posterior predictive checks on the variability in the posterior estimates for the lek count data to ensure that 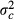 was accurately able to reflect the variability observed in the true data. The Bayesian p-value to evaluate the observed versus expected variance shows that the posterior estimates exhibit similar levels of variability as do the lek count data (*P*-value = 0.563, Gelman et al. 2013).

**Figure 5:**
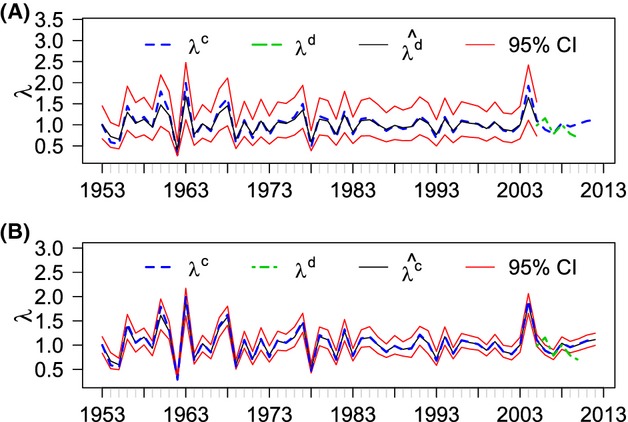
(A) Posterior predicted values for the population growth rate from the demographic data (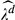) when demographic data were not available (1953–2004). (B) Posterior means for the population growth rate from the lek count data (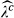). Both for data set from 1953 to 2012 with 95% credible intervals. The growth rates calculated from the lek count data (*λ*^*c*^, from 1953 to 2012) and demographic data (*λ*^*d*^, from 2005 to 2010) are shown for comparison.

We fit the constant growth rate integrated model to the entire time series (1953–2012) with the same priors (Table 1). We did not fit the linear or quadratic models as the longer time frame may require a more complex time series analysis to adequately model the data, and the longer data set was likely too imprecise to fit these models. The plot of the posterior predicted values for the demographic growth rates (Fig. 5) shows the values that would be expected for population growth rate based on the integrated model. The average growth rate from 1953 to 2012 is near stable (0.992) but with considerable error (95% CI 0.663, 1.437).

**Figure 6:**
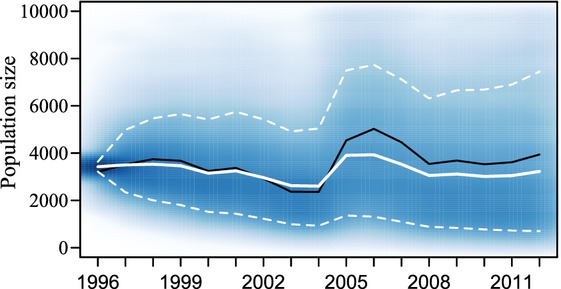
Estimated population sizes by year generated from the Markov chain Monte Carlo (MCMC) simulations for Gunnison sage-grouse in Gunnison Basin, Colorado. The darker shades represent the population sizes generated most often in the simulations. The solid white line is the posterior mean values of population size. The dashed white lines are the 90% credible intervals for the posterior population estimates. The black line represents the population estimate calculated directly from the lek count data.

Based on the posterior predictive estimates for population growth rate, we obtained population size projections for each year in the study. There is considerable variability in the population size predictions, but in general, the predicted population sizes are relatively stable with just over half of the simulations having a lower population size in 2012 than in 1996 (Fig. 6). The lek count data are often used not only as a population indicator, but also as a population estimator based on adjusting for the number of males assumed to be on leks and the expected ratio of males to females. Although population estimates from lek counts are highly criticized (e.g., Walsh et al. 2004), we displayed these estimates here for comparison (Fig. 6).

## Discussion

A primary goal of our study was to evaluate the relationship between the two sources of data available for GUSG: demographic estimates of fecundity and survival, and population survey estimates from long-term lek count data. A unique challenge of this integration is that the two types of models do not share direct parameters. The only common element between the models is a derived parameter of population growth. The dominant eigenvalue from a Leslie matrix estimates population growth (under a stable age distribution; Caswell 2001). Additionally, the rate of population changes from one time step to the next (*M*_*t*+1_/*M*_*t*_) estimates population growth; this method is applicable to population count data like that of the lek counts from our study (Link and Sauer 2002; Sauer and Link 2002).

Although these different estimators of growth rate come from different techniques and different segments of the population, they should be related. Growth rates from matrix calculations represent the asymptotic growth rate achieved when the population has reached a stable state (Caswell 2001), and are not precisely equivalent to the single time step growth rates (*M*_*t*+1_/*M*_*t*_). However, we calculated the matrix growth rates based on year-specific demographic parameters. If we assume the populations were close to the stable age distribution, these should approximate the year-specific growth rate for females. We are thus linking these matrix estimates of growth rate to those from the population count data. We recognize that the growth rates may not be identical, especially given that the demographic data are based on a female driven model and the population count data are only of males. Therefore, we assumed a linear relationship between the log of the year-specific growth rates (equation 9). This evaluation, based on the more reliable 1996–2012 time series, suggests that lek count estimates of population growth are typically biased high relative to demographic estimates and exhibit extreme high values that are not realistic based on demographic analysis.

Growth rates from count data are subject to more extreme values due to the strong influence of random error on count data. Similarly, growth rates from extensive demographic studies on the related greater sage-grouse (*Centrocercus urophasianus*) showed considerably less variability than the lek count growth rates we observed (Dahlgren 2009; Taylor et al. 2012). This difference in growth rate variability supports our finding of more extreme values in the lek count data (represented by parameter ‘b’ > 1 in equation 9) and suggests that the difference is not simply a result of the relatively short time frame for our demographic study. Dahlgren (2009) found that lek count estimates of population growth are routinely higher than estimates from population modeling. Potential sources of positive bias in lek count growth rates are detailed below but may include a selection bias (leks with many birds are counted more that those with fewer birds). In our study, growth rates from the count data also tended to be higher than those from the demographic data (parameter ‘a’ > 0 in equation 9) but the credible intervals for the difference overlapped zero (Table 3).

Each of the available data sources for GUSG has its strengths and weaknesses. The lek count data are long running and relatively inexpensive to collect as they depend heavily on volunteer support, which also leads to community involvement and awareness (Bell et al. 2008). However, these data come with various drawbacks. Long-term data can be difficult to manage consistently over time, especially with frequent turnover of people in charge of the data set and transitions from different data management techniques over the past 60 years. Additionally, there are drawbacks of data collection that requires a multitude of observers, as observers vary in their ability to detect birds (Sauer et al. 1994). These problems lead to uncertainty in the data.

Lek count utility has been questioned based on the fact that lek counts are inconsistent within a year (Connelly et al. 2003; Walsh et al. 2004; Gunnison Sage-Grouse Rangewide Steering Committee 2005), detectability is not accounted for (Walsh et al. 2004, 2010), potential problems with observer bias may exist (Walsh et al. 2004), survey effort may not be consistent among years and/or spatial variability may be present (Connelly et al. 2003; Broms et al. 2010). The lek count data have potential as a population indicator but the extreme values and high variability suggest that caution should be used when drawing conclusions solely from these data. Through the use of this integrated model, the estimated population growth rates should be less extreme and converge closer to the mean for the growth rates (equation 5). Our study shows the ability for this type of modeling to achieve more precise estimates by combining the data sources.

The demographic data are intensive and statistically stronger, but only span a relatively small time series (2005–2010). Therefore, there is potential for bias in estimates of population viability that are based on a small sample size (Doak et al. 2005). Population projection models based on these demographic data (Davis 2012) suggest that the GUSG have been declining. The lek count data also suggest that GUSG were declining in the Gunnison Basin over the 6 years of the demographic study (2005–2010, Fig. 2). However, lek count data have a longer temporal extent than the demographic data and show that the population exhibited a considerable increase just prior to the demographic study being initiated (Fig. 2). The integrated model is designed to enable the evaluation of population growth based on a larger time series to help avoid misleading results from the small time series. Our study shows that the population growth rate for GUSG is variable but has not changed significantly in the time frame examined (Fig. 3). On average from 1996 to 2012, the growth rate was near stable (*λ* = 0.988, 95% CI: 0.893, 1. 079).

By modeling the two data sets together and comparing models with constant, linear, and quadratic trends, we were able to evaluate the changes in growth rate. These data support the model with no change in growth rate. Although the demographic data are statistically stronger than the lek count data, there are limitations to this data. Sampling for the demographic data is not truly random and may have an inherent bias. Additionally, the fact the demographic data were observed during a decline might result in a link between the two data sources that is negatively biased. It is unfortunate that the demographic study began the year after the considerably high growth rate in 2004 was observed. This would have provided a comparison for the population growth rates in a good year.

Another objective of our study was to evaluate the population size estimates and projections over time under the integrated modeling approach. The population index data for this time frame suggest a slightly positive trend (Fig. 6). However, according to the evaluation of the posterior population growth rates over the past 16 years, one would expect a population that has varied over time and declines slightly (due to the growth rate estimate slightly less than 1), which the population projections depict (Fig. 6). We also applied this integrated model to the longer, more variable time series available for this species (1953–2012). However, the variability and potential bias in these historic data make it unlikely that we have the precision we need to evaluate changes in this longer data set; therefore, we do not think it is valid to model this larger time frame.

There are many factors that contribute to the variability in the historic data (described above). The total number of leks counted each year has not been consistently recorded for Gunnison Basin; instead, a coarser measurement of “lek areas” has been recorded. While we are able to standardize the lek counts to some extent using “lek areas” as a measure of survey effort, the actual number of leks counted might not be completely addressed which would result in some biased estimates of growth rate change. This integrated modeling technique provides a valuable method to combine data sets that have no direct parameters in common.

Integrated modeling is a powerful and flexible statistical tool that can be adapted to many different scenarios. The advantage of integrated modeling for many wildlife studies is that it allows for the combination of different data types, by borrowing strength from more rigorous studies and adding longevity to sparse data (Besbeas et al. 2002). This is particularly advantageous for rare or declining species in which there is often a paucity of data. Our study demonstrates a novel method that allows for two data types to be formally linked through a derived parameter in a statistically rigorous manner. This is an increase in the flexibility currently demonstrated in the literature for Bayesian integrated population models. Additionally, being able to estimate the relationship between these parameters directly in the integrated model adds versatility that could have wide applications in wildlife data analysis. Our estimates of population growth for GUSG are corrected for potential small sample size bias in the demographic data and reduce the high variability present in the count data (Figs. 5) which is an improvement over the independent analysis of each data set.

## Supporting Information

**Appendix S1.** Parameter estimates and standard errors for Gunnison Sage-Grouse vital rates by year with vital rate means and process variance. **Appendix S2.** Covariance matrix for Gunnison Sage-Grouse vital rates calculated from six years of data. **Appendix S4.** High male lek count data of Gunnison Sage-Grouse from 1953–2012 in Gunnison Basin, Colorado, USA.

**Appendix S3.** Pseudo code to implement Markov Chain Monte Carlo simulations for integrated population model of Gunnison sage-grouse.
