# Supplementary material for: An integrated modeling approach to estimating Gunnison sage-grouse population dynamics: combining index and demographic data
Source: Ecol Evol. 2014 Oct 22;4(22):4247–57. doi: 10.1002/ece3.1290 (PMC4267864; doi:10.1002/ece3.1290)
Supplement: Supplementary file 1 [file ece30004-4247-SD1.pdf]

APPENDIX A. Parameter estimates and standard errors for Gunnison Sage-Grouse vital rates by year with vital rate means and process variance.

	2005		2006		2007		2008		2009		2010		Vital Rate	Process
Year	θ	SE	θ	SE	θ	SE	θ	SE	θ	SE	θ	SE	Mean	Variance
Nest Initiation Yearling	1.00	0.00	0.93	0.06	0.81	0.03	0.87	0.04	0.85	0.05	0.86	0.10	0.89	0.0015
Nest Initiation Adult	1.00	0.00	0.91	0.07	0.84	0.03	0.96	0.01	0.97	0.05	0.92	0.02	0.93	0.0017
Hen Success Yearling	0.25	0.10	0.58	0.10	0.35	0.08	0.61	0.11	0.40	0.09	0.37	0.07	0.43	0.0106
Hen Success Adult	0.25	0.10	0.61	0.10	0.29	0.08	0.70	0.11	0.45	0.09	0.39	0.07	0.45	0.0233
Chick Survival	0.47	0.02	0.42	0.02	0.37	0.02	0.32	0.01	0.27	0.02	0.22	0.02	0.34	0.0084
Juvenile Survival	0.73	0.12	0.53	0.10	0.39	0.08	0.38	0.06	0.28	0.06	0.18	0.06	0.42	0.0307
Yearling Female Survival	0.75	0.11	0.61	0.11	0.56	0.11	0.56	0.12	0.72	0.09	0.61	0.11	0.63	0.0005
Yearling Male Survival	0.73	0.18	0.56	0.21	0.52	0.23	0.52	0.23	0.68	0.18	0.57	0.22	0.60	0.0037
Adult Female Survival	0.71	0.12	0.56	0.11	0.50	0.11	0.50	0.11	0.68	0.09	0.56	0.10	0.58	0.0004
Adult Male Survival	0.52	0.19	0.31	0.18	0.27	0.16	0.27	0.17	0.45	0.18	0.33	0.17	0.36	0.0019
½ Clutch Size	2.93	0.00	2.93	0.00	2.93	0.00	2.93	0.00	2.93	0.00	2.93	0.00	2.93	0.0000

APPENDIX B. Covariance matrix for Gunnison Sage-Grouse vital rates calculated from six years of data.

	NIY	NIA	HSY	HSA	SC	SJ	SYF	SYM	SAF	SAM
Nest Initiation Yearling (NIY)	0.004									
Nest Initiation Adults (NIA)	0.002	0.003								
Hen Success Yearling (HSY)	-0.001	-0.001	0.016							
Hen Success Adult (HSA)	-0.001	0.001	0.020	0.027						
Chick Survival (SC)	0.003	0.000	-0.001	-0.002	0.006					
Juvenile Survival (SJ)	0.008	0.001	-0.003	-0.006	0.013	0.029				
Yearling Female Survival (SYF)	0.003	0.003	-0.005	-0.005	0.001	0.003	0.005			
Yearling Male Survival (SYM)	0.003	0.003	-0.006	-0.007	0.001	0.004	0.006	0.006		
Adult Female Survival (SAF)	0.003	0.003	-0.006	-0.006	0.001	0.003	0.006	0.006	0.006	
Adult Male Survival (SAM)	0.004	0.004	-0.008	-0.008	0.002	0.005	0.007	0.008	0.007	0.009

*Clutch size was not calculated annually and thus is not included in the covariance matrix

APPENDIX D. High male lek count data of Gunnison Sage-Grouse from 1953-2012 in Gunnison Basin, Colorado, USA. The number of lek areas counted each year are provided.

Year	High male count	Number of lek areas counted
1953	617	5
1954	386	5
1955	136	3
1956	404	0
1957	355	10
1958	335	9
1959	486	11
1960	413	10
1961	296	4
1962	583	6
1963	338	12
1964	617	11
1965	468	12
1966	445	11
1967	378	11
1968	530	11
1969	862	11
1970	486	11
1971	539	11
1972	303	9
1973	228	6
1974	200	7
1975	404	0
1976	431	13
1977	472	12
1978	706	12
1979	307	11
1980	370	11
1981	379	10
1982	264	10
1983	385	11
1984	381	14
1985	434	14
1986	364	10
1987	402	11
1988	438	14
1989	277	9
1990	416	15
1991	206	8
1992	439	14
1993	197	6
1994	245	11
1995	449	17
1996	587	27
1997	645	27
1998	685	27
1999	723	28
2000	638	28
2001	712	29
2002	617	29
2003	500	29
2004	498	29
2005	958	29
2006	1061	29
2007	941	29
2008	748	29
2009	778	29
2010	745	29
2011	763	29
2012	832	29
