# Supplementary material for: An integrated modeling approach to estimating Gunnison sage-grouse population dynamics: combining index and demographic data
Source: Ecol Evol. 2014 Oct 22;4(22):4247–57. doi: 10.1002/ece3.1290 (PMC4267864; doi:10.1002/ece3.1290)
Supplement: Supplementary file 3 — Appendix S3. Pseudo code to implement Markov Chain Monte Carlo simulations for integrated population model of Gunnison sage-grouse. [file ece30004-4247-SD3.pdf]

Supplemental Materials for An Integrated Modeling Approach to Estimating Gunnison Sage-Grouse Population Dynamics: Combining Index and Demographic Data by Amy J. Davis, Mevin Hooten, Michael L. Phillips, Paul F. Doherty Jr.

May 20, 2013

Appendix C

To sample from the joint posterior distribution for this integrated model we iteratively sampled from the full-conditional distributions. We ran three different models (constant, linear, and quadratic effects on the average growth rate); the most complex of those models (quadratic) is shown here. In our model that entailed sampling from the full conditionals of: λ_t^c, b_0, b_1, b_2, σ_r^2, a, b, and σ_λ^2, sequentially for t=1,...,T. The full conditional distribution for λ_t^c is the only non-conjugate distribution and therefore must be sampled using a Metropolis-Hastings step. The proportional full-conditionals for the model parameters are as follows:

$$[log(\lambda_t^c) \mid \bullet] \propto \prod_{t=2}^{T} Pois(M_t \mid \omega_t exp(\lambda_t^c + log(M_{t-1}/\omega_{t-1}))) \times N(log(\lambda_t^c) \mid b_0 + b_1 * t + b_2 * t^2, \sigma_r^2) \times$$

$$\prod_{t \in \tau} N(log(\lambda_t^d) \mid a + b * log(\lambda_t^c), \sigma_\lambda^2) \quad (1)$$

$$[b_0 \mid \bullet] \propto \prod_{t=2}^{T} N(log(\lambda_t^c) \mid b_0 + b_1 * t + b_2 * t^2, \sigma_r^2) \times N(b_0 \mid \mu_\mu, \sigma_\mu^2) \quad (2)$$

$$[b_1 \mid \bullet] \propto \prod_{t=2}^{T} N(log(\lambda_t^c) \mid b_0 + b_1 * t + b_2 * t^2, \sigma_r^2) \times N(b_1 \mid 0, \sigma_{b1}^2) \quad (3)$$

$$[b_2 \mid \bullet] \propto \prod_{t=2}^{T} N(log(\lambda_t^c) \mid b_0 + b_1 * t + b_2 * t^2, \sigma_r^2) \times N(b_2 \mid 0, \sigma_{b2}^2) \quad (4)$$

$$[\sigma_r^2 \mid \bullet] \propto \prod_{t=2}^{T} N(log(\lambda_t^c) \mid b_0 + b_1 * t + b_2 * t^2, \sigma_r^2) \times IG(\sigma_r^2 \mid \gamma_1, \gamma_2) \quad (5)$$

$$[a \mid \bullet] \propto \prod_{t \in \tau} N(log(\lambda_t^d) \mid a + b * log(\lambda_t^c), \sigma_\lambda^2) \times N(a \mid 0, \sigma_a^2) \quad (6)$$

$$[b \mid \bullet] \propto \prod_{t \in \tau} N(log(\lambda_t^d) \mid a + b * log(\lambda_t^c), \sigma_\lambda^2) \times N(b \mid 1, \sigma_b^2) \quad (7)$$

$$[\sigma_\lambda^2 \mid \bullet] \propto \prod_{t=2}^{T} N(log(\lambda_t^d) \mid a + b * log(\lambda_t^c), \sigma_\lambda^2) \times IG(\sigma_\lambda^2 \mid r, q) \quad (8)$$

The product Normal for the log(λ_t^c) for t=2,...,T is included in the full conditionals for b_0, b_1, b_2, σ_r^2, and log(λ_t^c) because log(λ_t^c) is a function of those parameters. The product Normal for the log(λ_t^d) for $t \in \tau$ (τ is the range of years where demographic data are available)is included in the full condistions for a, b, σ_λ^2, and log(λ_t^d), because log(λ_t^d) is a function of those parameters. The steps below can be used to impliment the Gibbs sampling protocol to sample from the posterior distribution for this model.

1. Set initial values for model parameters: $\lambda_2^{c(0)}$, $b_0^{(0)}$, $b_1^{(0)}$, $b_2^{(0)}$, $\sigma_r^{2(0)}$, $a^{(0)}$, $b^{(0)}$, and $\sigma_\lambda^{2(0)}$. Using the initial value for $\lambda_2^{c(0)}$ to run the model foward to generate initial values for $\lambda_t^{c(0)}$ for t=3,...,T.

2. Sample $\lambda_1^{c(*)}$ from a proposal distribution, e.g., $[\lambda_1^{c(*)} \mid \lambda_1^{c(j-1)}]$, where j is the MCMC iteration. Run the process forward given the initial conditions to obtain values for $\lambda_t^{c(*)}$ for t=3,...,T. If the proposal distribution is choosen to be symmetric with respect to $\lambda_1^{c(*)}$ and $\lambda_1^{c(j-1)}$, (e.g., N(log($\lambda_1^{c(*)}$) $\mid log(\lambda_1^{c(j-1)}), \sigma^2_{l.tune}$), with tuning parameter $\sigma^2_{l.tune}$), then let $\lambda_1^{c(*)} = \lambda_1^{c(j-1)}$ if $p^*_\lambda > \nu$; otherwise let $\lambda_1^{c(j)} = \lambda_1^{c(j-1)}$. Where $\nu \sim Uniform(0,1)$ and

$$p^*_\lambda = \frac{\prod_{t=2}^{T} Pois(M_t \mid \omega_t e^{(\lambda_t^c + log(\frac{M_{t-1}}{\omega_{t-1}}))}) \times N(log(\lambda_t^c) \mid b_0 + b_1 * t + b_2 * t^2, \sigma_r^2)}{\prod_{t=2}^{T} Pois(M_t \mid \omega_t e^{(\lambda_t^c + log(\frac{M_{t-1}}{\omega_{t-1}}))}) \times N(log(\lambda_t^c) \mid b_0 + b_1 * t + b_2 * t^2, \sigma_r^2)} \times \frac{\prod_{t\in\tau} N(log(\lambda_t^d) \mid a + b * log(\lambda_t^c), \sigma^2_\lambda)}{\prod_{t\in\tau} N(log(\lambda_t^d) \mid a + b * log(\lambda_t^c), \sigma^2_\lambda)} \quad (9)$$

3. The remaining parameters (b_0, b_1, b_2, σ_r^2, a, b, and σ^2_λ) are modeled with conjugate prior distributions and therefore, can be sampled sequentially as follows:

For t=2,...,T

$$b_0^{(j)} \sim N\left(\frac{\frac{\mu_\mu}{\sigma_\mu^2} + \frac{1}{\sigma_r^2}\sum_{t=2}^{T}(\log(\lambda_t^c) - b_1 * t - b_2 * t^2)}{(\frac{t-1}{\sigma_r^2} + \frac{1}{\sigma_\mu^2})^{-1}}, \left(\frac{t-1}{\sigma_r^2} + \frac{1}{\sigma_\mu^2}\right)^{-1}\right) \tag{10}$$

$$b_1^{(j)} \sim N\left(\frac{\frac{1}{\sigma_r^2}\sum_{t=2}^{T} t * (\log(\lambda_t^c) - b_0 * t - b_2 * t^2)}{\left(\frac{\sum_{t=2}^{T} t^2}{\sigma_r^2} + \frac{1}{\sigma_{b1}^2}\right)^{-1}}, \left(\frac{\sum_{t=2}^{T} t^2}{\sigma_r^2} + \frac{1}{\sigma_{b1}^2}\right)^{-1}\right) \tag{11}$$

$$b_2^{(j)} \sim N\left(\frac{\frac{1}{\sigma_r^2}\sum_{t=2}^{T} t^2 * (\log(\lambda_t^c) - b_0 * t - b_1 * t)}{\left(\frac{\sum_{t=2}^{T} t^4}{\sigma_r^2} + \frac{1}{\sigma_{b2}^2}\right)^{-1}}, \left(\frac{\sum_{t=2}^{T} t^4}{\sigma_r^2} + \frac{1}{\sigma_{b2}^2}\right)^{-1}\right) \tag{12}$$

$$\sigma_r^{2(j)} \sim InverseGamma\left(\gamma_1 + \frac{1}{2}\sum_{t=2}^{T}\left(log(\lambda_t^c) - b_0 - b_1 * t - b_2 * t^2\right)^2, \frac{t-1}{2} + \gamma_2\right) \tag{13}$$

For t$\in \tau$

$$a^{(j)} \sim N\left(\frac{\frac{1}{\sigma_\lambda^2}\sum_{t\in\tau}(log(\lambda_t^d) - b * log(\lambda_t^c))}{\left(\frac{t_\tau}{\sigma_a^2} + \frac{1}{\sigma_\lambda^2}\right)^{-1}}, \left(\frac{t_\tau}{\sigma_a^2} + \frac{1}{\sigma_\lambda^2}\right)^{-1}\right) \tag{14}$$

$$b^{(j)} \sim N\left(\frac{\frac{1}{\sigma_\lambda^2}\sum_{t\in\tau}(log(\lambda_t^d) * log(\lambda_t^c) - a)}{\left(\frac{t_\tau}{\sigma_b^2} + \frac{1}{\sigma_\lambda^2}\right)^{-1}}, \left(\frac{t_\tau}{\sigma_b^2} + \frac{1}{\sigma_\lambda^2}\right)^{-1}\right) \tag{15}$$

$$\sigma_\lambda^{2(j)} \sim InverseGamma\left(r + \frac{1}{2}\sum_{t\in\tau}(log(\lambda_t^d) - a - b * log(\lambda_t^c))^2, \frac{t_\tau}{2} + q\right) \tag{16}$$

4. Posterior predictive values for λ^c can be generated by sampling from the conditional posterior values: $log(\tilde{\lambda_t^d}) \sim N\left(a^{(j)} + b^{(j)} * log(\lambda_t^{c(j)}), \sigma_\lambda^{2(j)}\right)$. We can calculate $\tilde{\lambda_t^d} \forall t$.

5. Once convergence is attained (i.e., $j > b$, for a burn-in period b), parameter values can be stored, thined to remove auto-correlation, and summary statistics can be calculated. The process should be repeated (steps 2-4) until a sufficiently large sample has been obtained in order to approximate the posterior distribution.
